# Molecular, Physiological, and Histopathological Insights into the Protective Role of *Equisetum arvense* and *Olea europaea* Extracts Against Metronidazole-Induced Pancreatic Toxicity

**DOI:** 10.3390/life15121907

**Published:** 2025-12-13

**Authors:** Manal R. Bakeer, Maha M. Rashad, Asmaa A. Azouz, Rehab A. Azouz, Abdulmajeed Fahad Alrefaei, Sultan F. Kadasah, Mohamed Shaalan, Alaa M. Ali, Marwa Y. Issa, Salma I. El-Samanoudy

**Affiliations:** 1 Physiology Department, Faculty of Veterinary Medicine, Cairo University, P.O. Box 12211, Giza 12613, Egypt; 2 Biochemistry and Molecular Biology Department, Faculty of Veterinary Medicine, Cairo University, Giza 12613, Egypt; maha_mansour@live.com; 3 Pharmacology Department, Faculty of Veterinary Medicine, Cairo University, Giza 12613, Egypt; asma_azoz@hotmail.com; 4 Toxicology and Forensic Medicine Department, Faculty of Veterinary Medicine, Cairo University, Giza 12613, Egypt; 5 Department of Biology, Jamoum University College, Umm Al-Qura University, Makkah 21955, Saudi Arabia; afrefaei@uqu.edu.sa; 6 Department of Biology, Faculty of Science, University of Bisha, Bisha 67714, Saudi Arabia; sukadasah@ub.edu.sa; 7 Pathology Department, Faculty of Veterinary Medicine, Cairo University, Giza 12613, Egypt; mohamedibrahim@cu.edu.eg (M.S.);; 8 Pharmacognosy Department, Faculty of Pharmacy, Cairo University, Giza 12613, Egypt

**Keywords:** metronidazole, acute pancreatitis, *Equisetum arvense* L., *Olea europaea* L., gene expressions, antioxidant, anti-inflammatory

## Abstract

**Background:** Acute pancreatitis is a significant global disease. This study investigated the phytochemical composition and potential protective effects of *Equisetum arvense* L. (horsetail) ethanol extract and *Olea europaea* L. (olive leaves) aqueous extract against metronidazole (MTZ)-induced pancreatic damage in rats. **Materials and Methods:** Rats were randomly divided into six groups: Group I (control) received saline; Group II (Metronidazole) received only MTZ (400 mg/kg). Group III (*Equisetum arvense* group) received *E. arvense* 100 mg/kg. Group IV (*Olea europaea*) received 400 mg/kg of *O. europaea*. Group V (MTZ + *E. arvense*) received both MTZ (400 mg/kg) and *E. arvense* (100 mg/kg). Group VI (MTZ + *O. europaea*) received MTZ (400 mg/kg) and *O. europaea* (400 mg/kg). All treatments were delivered daily via the oral route. After 60 days, serum amylase, lipase, protease, and glucose levels, oxidative parameters “malondialdehyde (MDA), catalase (CAT), mRNA relative expression of *pancreatic Pik3ca* (*phosphatidylinosi-tol-4*,*5-bisphosphate 3-kinase*, *catalytic subunit alpha*), *AKT* (*AKT Serine/Threonine Kinase 1*), *Nrf-2* (*Nuclear factor*, *erythroid 2-like 2*), *TNFα* (*tumor necrosis factor alpha*), and *IL-1β* (*interleukin-1 beta genes*, an apoptotic marker “caspase-3,” and histopathological changes were estimated. **Results:** HPLC analysis revealed that horsetail extract contained caffeic acid, catechin, rutin, and kaempferol, while olive leaf extract was dominated by oleuropein. MTZ administration significantly elevated serum levels of pancreatic enzymes (lipase, amylase, and protease) and glucose and increased oxidative stress markers, such as MDA, while reducing catalase (CAT) activity. Co-treatment with MTZ and horsetail, or MTZ and olive extracts, mitigated these effects, especially horsetail, which restored CAT levels and reduced MDA concentrations. qPCR analysis showed MTZ upregulated inflammatory genes (*TNFα*, *IL-1β*) and downregulated antioxidant and survival-related genes (*Pik3ca*, *AKT*, *Nrf-2*). Horsetail co-treatment significantly reversed these gene expression patterns. Histopathological and immunohistochemical analyses confirmed MTZ-induced pancreatic tissue degeneration and increased cleaved caspase-3 expression, both of which were notably alleviated by horsetail extract. **Conclusions:** These findings highlight the superior protective efficacy of *Equisetum arvense* over *Olea europaea* in ameliorating MTZ-induced pancreatic toxicity, potentially through anti-inflammatory, antioxidant, and anti-apoptotic mechanisms.

## 1. Introduction

. Metronidazole, an antibiotic belonging to the nitroimidazole class, is extensively utilized to manage infections attributed to anaerobic bacteria and various protozoan species. It is widely marketed under the trade name Flagyl [1]. Despite its efficacy, metronidazole has been associated with several adverse effects, including headache, peripheral neuropathy, gastrointestinal disturbances, a metallic taste, urticaria, and, in rare cases, pancreatitis [2]. Clinically, metronidazole-induced pancreatitis is characterized by sudden and persistent abdominal pain, elevated pancreatic enzyme levels, and often occurs in conjunction with biliary tract disorders, which account for approximately 95% of reported cases [3].

For millennia, traditional medicinal herbs have been used across cultures for maintaining health and treating various ailments [4]. Among these, the olive tree (*Olea europaea* L.) has garnered attention for its health-promoting properties. Animal studies have demonstrated that olive leaf extract (OLE) possesses anti-atherogenic, anti-inflammatory, hypoglycemic, and hypocholesterolemic effects, largely attributed to its potent antioxidant activity. This activity is believed to result from the synergistic interaction of flavonoids, oleuropeosides, and substituted phenolic compounds, with total OLE showing greater antioxidant potential than vitamins C and E [5]. Oleuropein, the main bioactive component of olive leaves, is considered primarily responsible for these pharmacological benefits due to its notable in vitro antioxidant capacity [6].

Likewise, *Equisetum arvense* L. (field horsetail), a species of the Equisetaceae family, is well known for its abundance of phenolic constituents, including flavonoids and phenolic acids, which underlie its notable antioxidant and anti-inflammatory properties [7,8].

The current study aimed at exploring the mechanism underlying metronidazole-induced pancreatitis and to evaluate the potential protective effects of *Equisetum arvense* and olive leaf extracts against metronidazole toxicity. Additionally, the study examines changes in selected biochemical markers related to pancreatic function and oxidative stress in both plasma and pancreatic tissue.

## 2. Materials and Methods

### 2.1. Animals and Experimental Design

The study was conducted using 30 adult male albino Wistar rats weighing between 180 and 220 g, obtained from the Animal House Facility at the Faculty of Veterinary Medicine, Cairo University. The animals were maintained under standard laboratory conditions (22 ± 2 °C, 12-h light/dark cycle). 

All rats were kept in ventilated plastic cages, with five animals per cage, and were provided commercial dry pellets and free access to tap water for the entire duration of the experiment. A two-week acclimation period preceded the start of the study. Rats were randomly divided into six groups (n = 5) and all treatments were given orally daily utilizing intragastric intubation.

The rats were treated daily for 60 days as follows:

1. Group I (Control group): received distilled water.
2. Group II (Metronidazole): received MTZ (400 mg/kg) only.
3. Group III (*Equisetum arvense* group): received *E. arvense* (100 mg/kg).
4. Group IV (*Olea europaea*): received *O. europaea* (400 mg/kg)
5. Group V (MTZ + *Equisetum arvense* group): received MTZ (400 mg/kg) plus *E. arvense* (100 mg/kg).
6. Group VI (MTZ + *Olea europaea*): received MTZ (400 mg/kg) plus *O. europaea* (400 mg/kg).

Dose of *Equisetum arvense* according to Hegedus et al. [9].

Dose of *Olea europaea* extracts according to Al-Attar and Alsalmi [10],

Dose of MTZ according to Hassan et al. [11].

### 2.2. Drug

MTZ was purchased from Sigma-Aldrich Chemical Co. Livonia, MI, USA. It was used at a dose of 400 mg/kg [11].

### 2.3. Ethical Approval and ARRIVE Guidelines Compliance

All experimental procedures involving animals were conducted in accordance with relevant guidelines and regulations and approved by the Institutional Animal Care and Use Committee (IACUC) of Cairo University, Faculty of Veterinary Medicine (Vet CU 09092023780).

Furthermore, we confirm that all methods were carried out in accordance with the ARRIVE guidelines and the ethical standards of the institutional and national research committees.

### 2.4. Preparation of Plant Extract

Olive (*Olea europaea* L.) leaves were harvested in October 2023 at the Experimental Station of Medicinal Plants, Faculty of Pharmacy, Cairo University, Giza, Egypt.

Horsetail (*Equisetum arvense* L.) samples imported from Syria were obtained in October 2023 from the Haraz herbal shop in Cairo, Egypt.

The samples’ identities were validated at the Cairo University Faculty of Science herbarium.

*Olea europaea* leaves were gathered, washed, and dried in a hot air dryer at 40 °C, after which the dried leaves were pulverized with a grinder. The dried powdered leaves (about 1 kg) were infused with boiling water to make an olive leaf infusion. Prior to filtration, the mixture was allowed to cool to room temperature. The infusion was then lyophilized, yielding an olive leaf aqueous extract.

The air-dried powder of *Equisetum arvense* (2 kg) was extracted with 70% ethanol and then soaked in ethanol for one day, following which the ethanol was filtered, and the powder was macerated twice until exhausted. The ethanol extracts were mixed and evaporated under vacuum at a low temperature of 40 °C until dryness, yielding a dark green residue of horsetail extract.

The extracts were stored at −40 °C for subsequent characterization and biological testing.

### 2.5. Total Phenolic Content (TPC) and Total Flavonoid Content (TFC)

The Folin–Ciocalteu colorimetric method was adopted for the determination of total phenolic content (TPC) [12]. The absorbances of the resulting solutions were measured at 765 nm using a FluoStar Omega microplate reader (BMG LABTECH, Ortenberg, Germany). A standard calibration curve was employed for gallic acid, and TPC was expressed as mg gallic acid equivalent per gram of dried extract (mg GAE/g).

For the total flavonoid content (TFC), a colorimetric method using aluminum chloride was employed [13]. A standard calibration curve was constructed for quercetin concentrations. The absorbances of the resulting solutions were measured at 420 nm using a FluoStar Omega microplate reader (BMG LABTECH). Total flavonoids were expressed as milligrams of quercetin equivalents per gram of dried extract (mg QE/g). Each experiment was performed in triplicate.

### 2.6. HPLC Characterization of Horsetail Ethanol Extract and Olive Leaves Aqueous Extract

Sample characterizations were achieved by a Waters 2690 Alliance HPLC system equipped with a Waters 996 photodiode array detector, HPLC Inertsil C18 (5 µm, 4.6 × 250 mm). The mobile phase consisted of solvent (A): 0.1% orthophosphoric acid, and solvent (B): Methanol. A linear gradient elution program was employed as follows: 0–3 min, 95% A; then the gradient was programmed to reach 50% A at 50 min and then to 30% A at 55 min, then to 10% A at 75 min and was then returned to 95% at 76 min and kept at 95% A for 5 more min. The flow rate: 1 mL/min, injection volume: 20 μL, and column temperature: 25 °C. Absorbance perception was conducted at 280 nm. Identification of compounds was performed by comparing their retention times with those of standard solution of authentic substances: phenolic acids (gallic, ellagic, and caffeic) and chlorogenic acids and flavonoids (catechin, quercetin, kaempferol, and rutin) for horsetail and oleuropein for olive leaves.

### 2.7. Specimen and Tissue Preparation

Twenty-four hours following the final dosage, fasting blood samples were collected from each rat using the orbital sinus puncture method after anesthesia with an intraperitoneal injection of ketamine (90 mg/kg) and xylazine (10 mg/kg) [14]. Blood samples intended for serum analysis were collected without an anticoagulant, allowed to clot, and the resulting serum was stored at −20 °C for pancreatic enzymes analysis. Extra blood samples were collected in fluoride tubes for blood glucose assessment. The serum biochemical parameters were measured using enzymatic spectrophotometry as recommended by the manufacturer’s procedure (Spin react, Spain). After sample collection, all rats were humanely euthanized under anesthesia. All animals were euthanized using an intraperitoneal injection of sodium pentobarbital (200 mg/kg), a method consistent with the AVMA Guidelines for the Euthanasia of Animals (2020). This approach ensures rapid, humane, and painless death and is widely accepted for laboratory rodents. Each rat’s pancreas was promptly transferred into 10% buffered formalin for histological analysis. The remaining pancreas was frozen and preserved at −80 °C for future oxidative stress tests and gene expression.

### 2.8. Evaluation of Oxidative Stress Parameters

Catalase (CAT) activity was evaluated spectrophotometrically according to Fossati et al. 1980 [15]. The concentration of lipid peroxidation (MDA) in pancreatic homogenates was determined by monitoring Thiobarbituric acid reactive substance (TBARS) production with colorimetric kits.

Tissue specimens from the tissue homogenates were used for the measurement of malondialdehyde (MDA) activity and CAT enzyme activity using a colorimetric method as per the directions of the manufacturer’s kits (Biodiagnostic, Cairo, Egypt).

### 2.9. qPCR Analysis of Pik3ca, AKT, Nrf-2, TNFα, and IL-1β Genes

Quantitative real-time PCR was used to evaluate relative pancreatic mRNA expression of *Pik3ca* (*phosphatidylinosi-tol-4*,*5-bisphosphate 3-kinase*, *catalytic subunit alpha*), *AKT* (*AKT Serine/Threonine Kinase 1*), *Nrf-2* (*Nuclear factor*, *erythroid 2-like 2*), *TNFα* (*tumor necrosis factor alpha*), *and IL-1β* (*interleukin-1 beta*) genes, with GAPDH (Glyceraldehyde3-phosphate dehydrogenase) as a housekeeping gene [16]. Total RNA extraction was performed on about 50 mg of pancreatic tissue using the Total RNA Extraction Kit (Vivantis, Malaysia) [17]. RNA content and purity were validated using the nanodrop technique [18]. RT-PCR was carried out using M-MuLV Reverse Transcriptase (NEB#M0253). qPCR analysis was carried out using SYBR green PCR Master Mix (Thermo Scientific, Cat. No. K0221) [19]. Primer sets were created using the free online program Primer3 (v. 0.4.0) https://primer3.ut.ee/. The primer sequences are shown in Table 1. Each qPCR was performed with three biological replicates, each of which was examined three times [20]. Negative controls without templates were added [21,22] and employed the comparative 2−ΔΔCT technique to calculate relative transcription levels [23].

### 2.10. Histopathological Examination

Pancreatic tissues fixed in formalin were routinely processed by graded alcohol dehydration, clearing in xylene, and embedding in paraffin. Serial sections of 4–5 μm thickness were then prepared from the paraffin blocks and stained with hematoxylin and eosin (H&E) following the method described by [24].

Paraffin slices were regularly deparaffinized, dehydrated, antigen-retrieved, and treated with 3% hydrogen peroxide (H_2_O_2_) for 30 min (to neutralize endogenous peroxidases). The slides were then cleaned three times with PBS and incubated with blocking serum for one hour. The samples were then incubated overnight at 4 °C with one of the primary antibodies, namely rabbit monoclonal anti-rat caspase 3, at dilutions of 1:100. The primary antibodies utilized in this study were acquired from Abcam, a renowned supplier based in Cambridge, Massachusetts, United States. After the washing process using phosphate-buffered saline (PBS), the sections underwent an incubation period of 30 min at ambient temperature. In this incubation period, a secondary antibody obtained from Dako Corp. (Glostrup, Denmark) was employed at a dilution ratio of 1:200. Furthermore, a combination of streptavidin and alkaline phosphatase (also acquired at Dako Corp.) was administered at a dilution ratio of 1:200. The utilization of diaminobenzidine (DAB) as a chromogen was implemented in order to enhance the visualization of antibody binding sites. Following that, the samples underwent a washing process using phosphate-buffered saline (PBS) and were subsequently counterstained with Hematoxylin for a period of 2 to 3 min. During the subsequent stage, the samples were subjected to dehydration through a sequential treatment involving ethanol solutions with progressively higher concentrations. Following this, the specimens underwent two successive immersions in xylene for a period of 5 min each, at ambient temperature, with the aim of attaining optical transparency. The samples were then processed and viewed under a high-resolution light microscope. The positive brown area for each marker’s expression was quantified by calculating the proportion of area in seven high-power microscopic fields with image analysis software (Image J, 1.46a, NIH, Bethesda, MD, USA).

### 2.11. Statistical Analysis

All quantitative data were examined with SPSS version 18.0 for Windows. The data were provided as mean ± SE (standard error). A one-way analysis of variance was used to compare means from several groups, followed by the Duncan test. Statistical significance was determined at *p* < 0.05.

## 3. Results

### 3.1. HPLC Characterization of Horsetail Ethanol Extract and Olive Leaves Aqueous Extract

**Figure 1:**
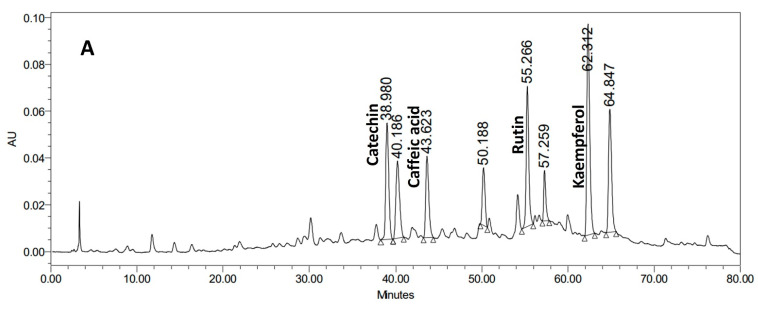

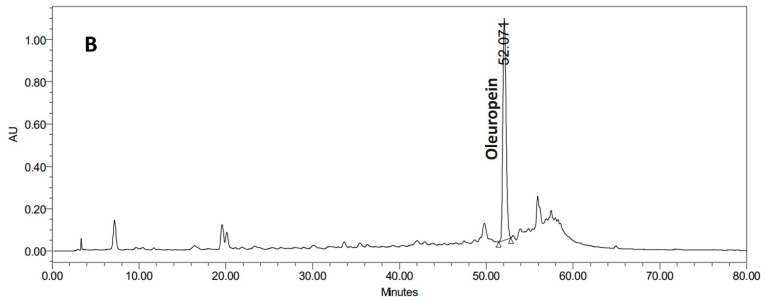
Representative HPLC chromatogram of horsetail ethanol extract (**A**) and olive leaves aqueous extract (**B**) showing the identified standard compounds.

Chromatographic analysis of selected phenolic compounds in the horsetail sample by HPLC allowed for the identification of four major compounds: caffeic acid, catechin, rutin, and kaempferol. Whereas the olive leaves sample revealed oleuropein as the major metabolite (Figure 1).

### 3.2. Serum Pancreatic Enzymes

**Figure 2:**
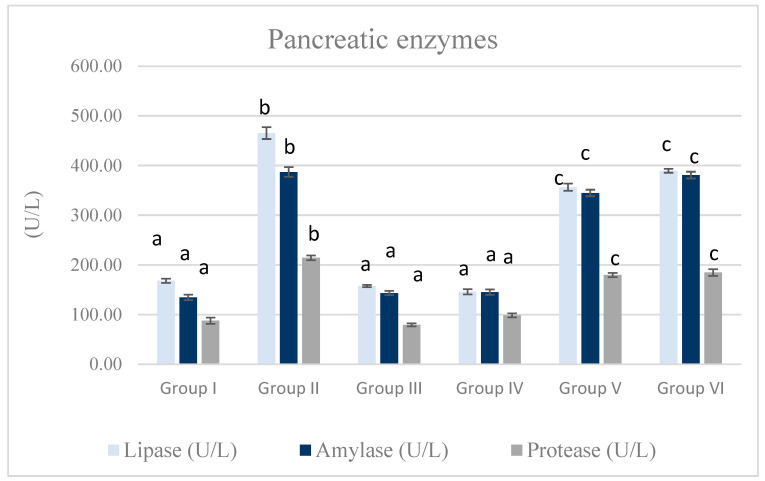
The effect of MTZ, *Equisetum arvense* L., and *Olea europaea* L. on serum lipase, amylase, and protease of rats. Data are represented as mean ± SE. Groups having different letters are significantly different from each other at *p* < 0.05. Groups having similar letters are non-significantly different from each other at *p* < 0.05.

As shown in Figure 2, the MTZ group revealed a significant increase in pancreatic enzymes (lipase, amylase, and protease) in comparison with the control, *Equisetum arvense*, and *Olea europaea* groups (*p* < 0.05). On the contrary, the MTZ cotreatment with *Equisetum arvense* L. succeeded in alleviating MTZ-induced drastic effects on the pancreatic enzymes compared to the MTZ group (*p* < 0.05).

### 3.3. Serum Glucose Level

**Figure 3:**
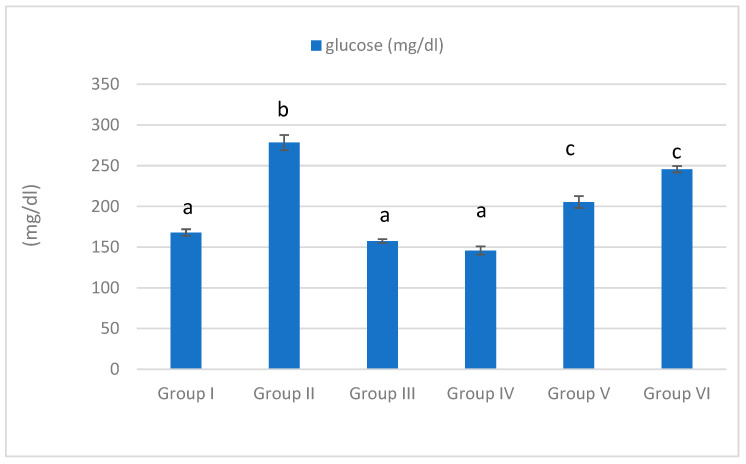
The effect of MTZ, *Equisetum arvense*, and *Olea europaea* on the glucose level of rats. Data are represented as mean ± SE. Groups having different letters are significantly different from each other at *p* < 0.05. Groups having similar letters are non-significantly different from each other at *p* < 0.05.

Our current results indicate that the MTZ treatment significantly increased glucose level in comparison with the control group (*p* < 0.05), as shown in Figure 3. On the other hand, MTZ cotreatment with *Equisetum arvense* and *Olea europaea* succeeded in significantly decreasing glucose level.

### 3.4. Antioxidant and Oxidative Stress

The TPC for *Olea europaea* leaves aqueous extract and *Equisetum arvense* 70% ethanol extract were 158.92 ± 3.73 and 118.18 ± 1.90 mg GAE/g dry extract, respectively.

The TFC for *Olea europaea* leaves aqueous extract and *Equisetum arvense* 70% ethanol extract were 101.20 ± 4.86 and 78.26 mg ± 2.53 QE/g dry extract, respectively.

**Figure 4:**
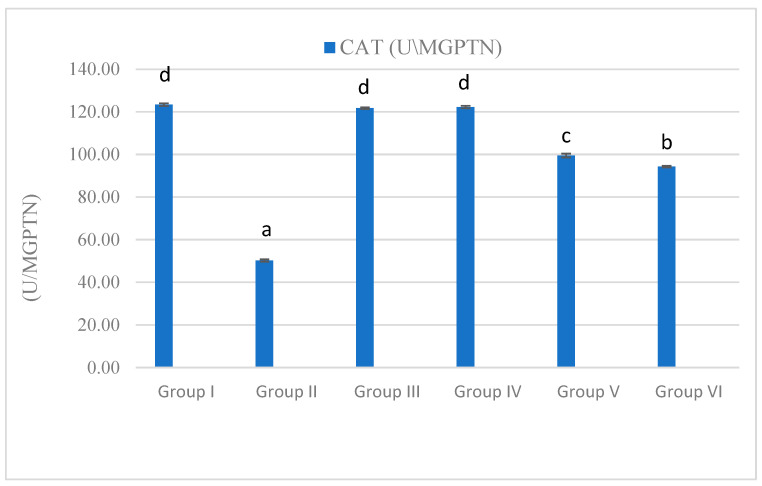
Effect on the catalase activity observed after treatment with *Olea europaea* leaf extract and *Equisetum arvense* ethanol extract at doses of 400 and 100 mg/kg, respectively, on metronidazole-induced pancreatitis in rats. Data are expressed as mean ± standard error (SE). Groups labeled with different letters indicate a statistically significant difference at *p* < 0.05, whereas groups sharing the same letter do not differ significantly at the same significance level.

**Figure 5:**
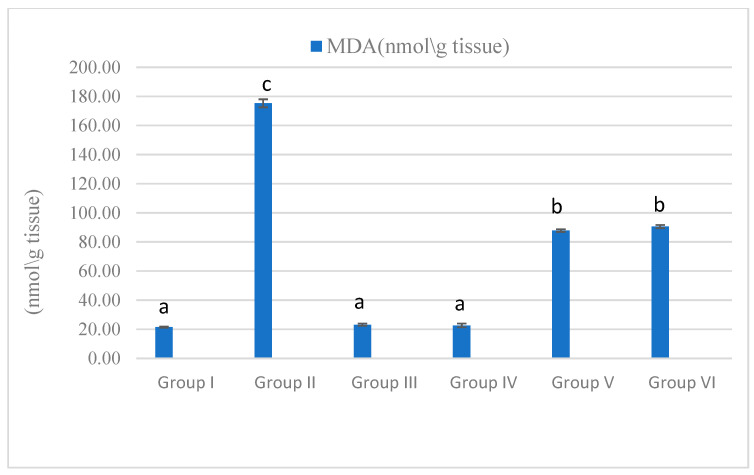
Effect on the MDA activity observed after treatment with *Olea europaea* leaf extract and *Equisetum arvense* ethanol extract at doses of 400 and 100 mg/kg, respectively, on metronidazole-induced pancreatitis in rats. Data are expressed as mean ± standard error (SE). Groups labeled with different letters indicate a statistically significant difference at *p* < 0.05, whereas groups sharing the same letter do not differ significantly at the same significance level.

The horsetail and olive leaves extract significantlyreduced the elevated levels of MDA in the MET group. Moreover, the MET group exhibited a significant decrease in the activity of the CAT enzyme, which was restored by the horsetail and olive leaf extracts (Figure 4 and Figure 5).

### 3.5. qPCR Analysis of Pik3ca, AKT, Nrf-2, TNFα, and IL-1β Genes

#### 3.5.1. Pancreatic mRNA Relative Expression of *TNFα* and *IL-1β* Genes

**Figure 6:**
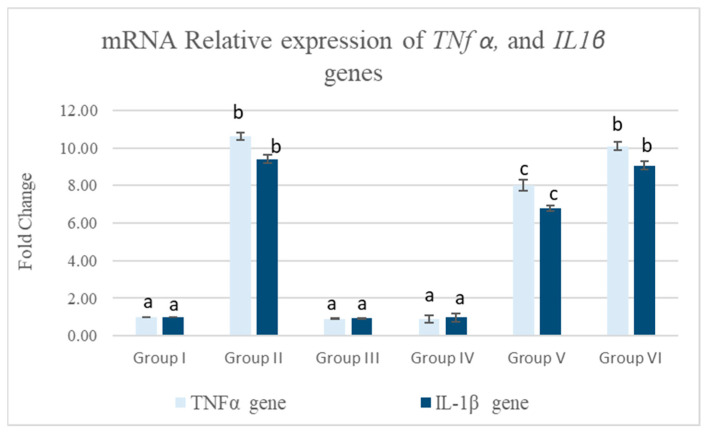
The effect of MTZ, *Equisetum arvense* L., and *Olea europaea* L. on *TNFα* and *IL-1β* gene expression in the pancreatic tissue of rats. Data are expressed as mean ± standard error (SE). Groups labeled with different letters indicate a statistically significant difference at *p* < 0.05, whereas groups sharing the same letter do not differ significantly at the same significance level.

As shown in Figure 6, the MTZ group revealed a significant upregulation in pancreatic mRNA-relative expressions of *TNFα* and *IL-1β* in comparison with the control group (*p* < 0.05). On the contrary, the MTZ cotreatment with *Equisetum arvense* L. succeeded in alleviating MTZ-induced drastic effects on the pancreatic expression of both genes compared to the MTZ group (*p* < 0.05).

#### 3.5.2. Pancreatic mRNA Relative Expression of *Pik3ca*, *AKT*, and *Nrf-2* Genes

**Figure 7:**
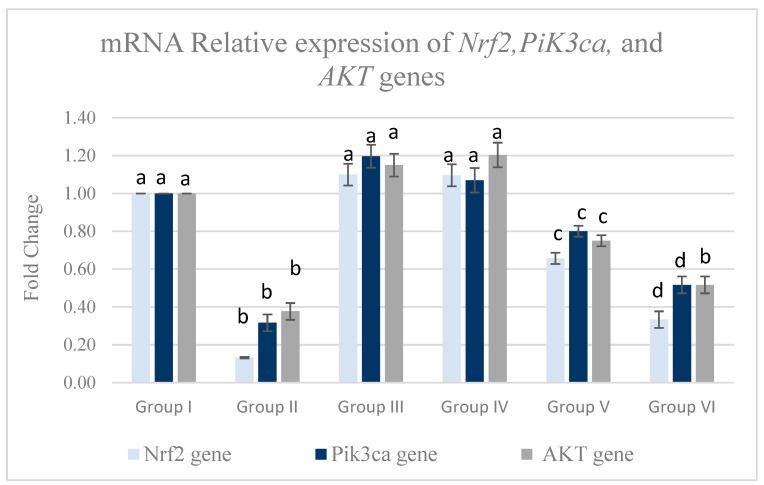
The effect of MTZ, *Equisetum arvense* L., and *Olea europaea* L. on *Pik3ca*, *AKT*, and *Nrf*-2 gene expression in the pancreatic tissue of rats. Data are expressed as mean ± standard error (SE). Groups labeled with different letters indicate a statistically significant difference at *p* < 0.05, whereas groups sharing the same letter do not differ significantly at the same significance level.

Our current results indicate that MTZ treatment significantly downregulated pancreatic mRNA relative expression of *Pik3ca*, *AKT*, and *Nrf2* genes in comparison with the control group (*p* < 0.05), as shown in Figure 7. On the other hand, MTZ cotreatment with *Equisetum arvense* L. succeeded in significantly downregulating pancreatic mRNA-relative expressions of *Pik3ca*, *AKT*, and *Nrf-2* genes.

### 3.6. Histopathological and Immunohistochemical Effects of Different Treatments on the Pancreatic Tissues

**Figure 8:**
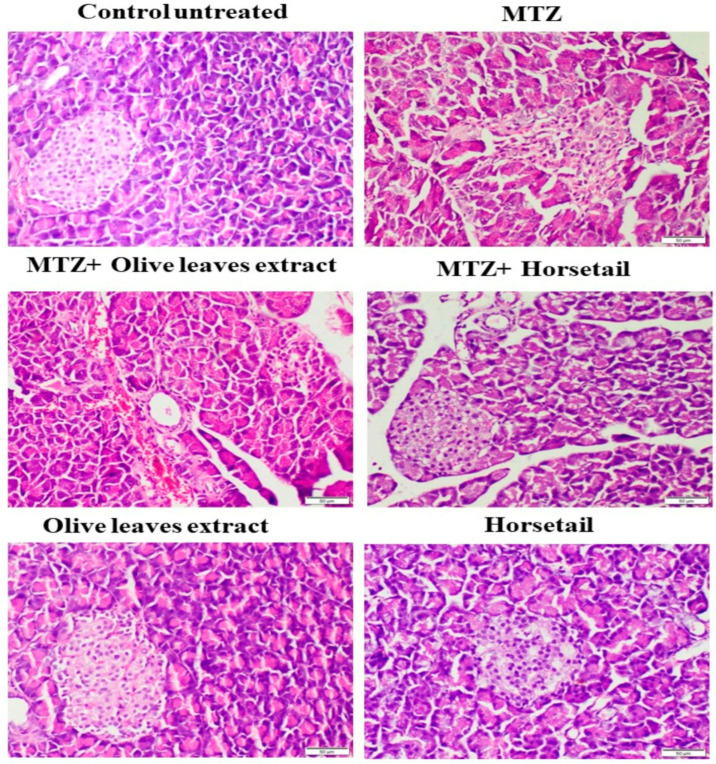
Microphotographs of H&E-stained pancreas tissue sections showing the effects of horsetail and olive oil administration in MTZ-induced diabetic rats. Untreated rats from the control group showed normal histological structure for both endocrine and exocrine glands of pancreatic tissue; MTZ-treated rats (control + ve) revealed a significant decrease in the size of islet cells, as well as a distortion of their shape, abnormal pancreatic architecture, as well as degeneration and atrophy of pancreatic lobes with widening of sinusoidal spaces. Mononuclear cell infiltration around interlobular ducts in some pancreatic regions and dilatation of blood vessels. Moreover, they showed vacuolation of cell populations, which could reach complete absence. The administration of the *Equisetum arvense* extract treatment to MTZ-treated rats (MTZ + Horsetail) revealed significant regeneration and restoration of the islet size with mild vacuolations in pancreatic acinar cells. MTZ mixed with olive leaves extract treatment group (MTZ + Olive) showed severe distortion of both endocrine and exocrine regions of pancreatic tissues, like those in the histological pictures of the MTZ group. Both the Olive and Horsetail groups revealed normal histological pancreatic architectures.

Untreated rats in the control group showed a normal histological structure for both the endocrine and exocrine glands of the pancreatic tissue. The histological sections of the MTZ-treated rats (control + ve) (Figure 8) revealed a significant decrease in the size of islet cells, as well as a distortion of their shape. Compared to normal groups, they revealed abnormal pancreatic architecture as well as degeneration and atrophy of pancreatic lobes with widening of sinusoidal spaces, mononuclear cell infiltration around inter-lobular ducts in some pancreatic regions, and dilatation of blood vessels. Additionally, histological sections of the MTZ-treated groups revealed that the vacuolation of cell populations could reach complete absence. The administration of the horsetail treatment to the MTZ-treated rats (MTZ + Horsetail) revealed significant regeneration and restoration of the islet size with mild vacuolations in pancreatic acinar cells (Figure 7). The MTZ mixed with olive treatment group (MTZ + Olive) showed a severe distortion of both endocrine and exocrine regions of the pancreatic tissues, like those in the histological pictures of the MTZ group. Both the Olive and Horsetail groups revealed normal histological pancreatic architectures.

**Figure 9:**
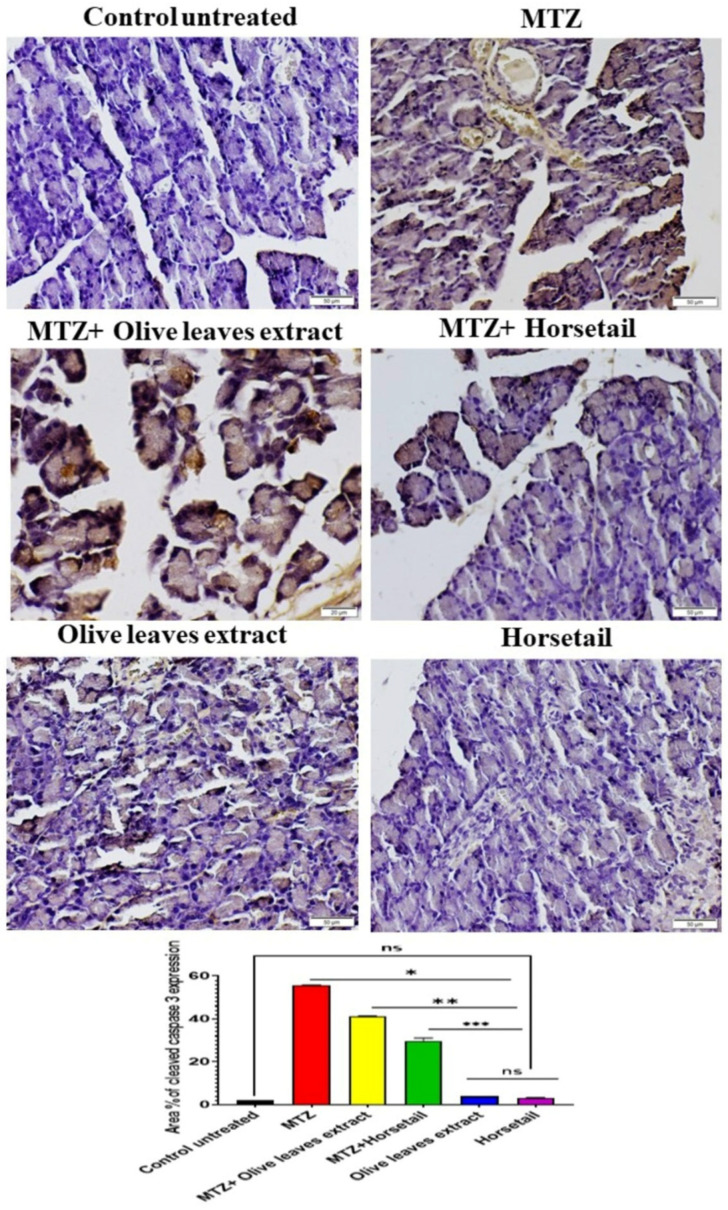
Area percentage of cleaved caspase expression in immune-stained pancreas tissue sections showing the effects of horsetail and olive oil administration in MTZ-induced diabetic rats. A pronounced and widespread manifestation of cleaved Caspase-3 was detected in the MTZ and MTZ + Olive leaf groups. The experimental group that received a combination of MTZ and horsetail extract (mg/kg) exhibited a statistically significant reduction in the expression of pancreatic cleaved caspase-3, as determined through analysis using image analysis software, when compared to the group that received only MTZ. There was no statistically significant difference observed among the untreated control group, the group treated with olive leaf extract, and the group treated with horsetail. (Statistical significance is indicated as follows: ns denotes statistically significant difference. * indicates a significate difference vs. control group; ** indicates a significate difference vs. MTZ group; and *** indicates a significate difference vs. MTZ + olive oil extract at *p* > 0.05).

Figure 9 depicts the immune expression of cleaved Caspase-3 in the pancreatic tissue. A pronounced and widespread manifestation of cleaved Caspase-3 was detected in the MTZ and MTZ+*Olea europaea* groups, while noticeably reduced expressions were identified in the pancreatic sections of all other treatment groups. The experimental group that received a combination of MTZ and *Equisetum arvense* extract (100 mg/kg) exhibited a statistically significant reduction in the expression of pancreatic cleaved caspase-3, as determined through analysis using image analysis software, when compared to the group that received only MTZ. There was no statistically significant difference observed among the untreated control group, the group treated with *Olea europaea* leaf extract, and the group treated with *Equisetum arvense* ethanol extract.

## 4. Discussion

Pancreatitis encompasses a wide spectrum of etiologies, severities, complications, and clinical outcomes. Among the less common drug-induced causes is metronidazole, a widely used antimicrobial agent [1]. Our current findings demonstrate that metronidazole (MTZ) administration significantly elevates serum amylase, lipase, and protease levels, likely due to oxidative stress, as evidenced by increased MTZ-related activity. These observations align with the report by [2], which described a threefold increase in serum amylase and/or lipase in a patient with acute pancreatitis following MTZ treatment.

The observed hyperglycemia may be attributed to impaired insulin secretion caused by oxidative damage to pancreatic β-cells. This is supported by Sura et al. [25], who reported similar effects. A plausible mechanism for MTZ-induced pancreatitis involves the generation of reactive oxygen species (ROS), leading to β-cell injury [26].

Our oxidative stress markers revealed a significant rise in malondialdehyde (MDA) and a marked reduction in catalase (CAT) activity. This may be due to MTZ undergoing redox cycling under aerobic conditions, producing harmful radicals such as hydrogen peroxide and superoxide, which damage pancreatic β-cells. Increased lipid peroxidation suggests oxidative injury to cellular membranes, contributing to reduced levels of endogenous antioxidants, including glutathione (GSH) [27,28]. Durairaj et al. [29] reported that diminished CAT activity could lead to the accumulation of ROS, further exacerbating oxidative stress. Similarly, Kumari and Singh [30] found elevated MDA levels and reduced CAT activity in MTZ-treated mice, confirming the pro-oxidative stress potential of MTZ. Furthermore, the concurrent increase in MDA and reduction in CAT levels may elevate hydrogen peroxide and its reactive derivative, hydroxyl radicals. However, contrasting evidence has indicated potential antioxidant effects of MTZ under specific conditions [31].

Our data also indicate that MTZ significantly upregulated the expression of pro-inflammatory markers (*TNF-α* and *IL-1β*) while downregulating *PI3K*, *AKT*, and *Nrf2* mRNA expression. Increased caspase-3 activity and inflammatory cell infiltration were confirmed by hematoxylin and eosin (H&E) staining. These results are consistent with El-Moslemany et al. [32], who demonstrated MTZ-induced *Nrf2* downregulation and oxidative stress in brain tissue. Maharati and Moghbeli [33] similarly found that MTZ administration resulted in oxidative stress and pancreatic inflammation.

The PI3K/AKT pathway is essential for cell survival, apoptosis regulation, and glucose metabolism [34]. Nrf2, a critical downstream transcription factor of this pathway, governs the expression of antioxidant, cytoprotective, and detoxifying enzymes through the antioxidant response element (ARE) [16]. The dysregulation of Nrf2 has been implicated in chronic inflammation, cancer, and aging-related diseases [35].

Interest in herbal therapies is increasing due to their natural origin, safety, and efficacy in managing diseases such as diabetes, cancer, and inflammatory conditions [36,37]. *Equisetum arvense* L. contains a rich profile of bioactive secondary metabolites, such as phenolics, flavonoids, terpenoids, sterols, alkaloids, and saponins, with proven antioxidant, anti-inflammatory, antidiabetic, and antimicrobial effects [38]. Phenolic and flavonoid compounds, in particular, act as potent ROS scavengers and have demonstrated significant antidiabetic activity [39,40].

In our study, treatment with *E. arvense* and *Olea europaea* leaf extracts significantly reduced pancreatic enzyme levels, blood glucose, and lipid peroxidation, while enhancing CAT activity. This effect is likely due to the antioxidant and anti-inflammatory properties of phytochemicals, particularly flavonoids [41]. It also highlighted the role of polyphenols in reducing lipid peroxidation. *E. arvense* extract effectively reduced MDA levels in rats subjected to CCl_4_-induced oxidative stress, supported by its phytochemical composition [42,43].

*Olea europaea* leaf extract also exhibited strong antioxidant and hypoglycemic effects, attributed to its high phenolic and flavonoid content. Aqueous extracts showed robust α-glucosidase inhibition, improved glycemic control, and repaired damage in pancreatic, hepatic, and renal tissues [5,6]. These properties support its use as an adjuvant or stand-alone therapy in type 2 diabetes management.

Pharmacological investigations have increasingly highlighted the therapeutic potential of *E. arvense* [44,45]. In our study, treatment with *E. arvense* extract in MTZ-treated rats led to a significant downregulation of *TNF-α* and *IL-1β* and an upregulation of PI3K, AKT, and Nrf2 expression. These effects may stem from the activation of the PI3K/AKT/Nrf2 signaling axis, which protects against ROS-induced cell damage [29]. The flavonoid isococitrin (quercetin-3-O-glucoside) found in *E. arvense* is also thought to play a key role in this process by modulating immune function and suppressing TNF-α and IFN-γ synthesis [46].

Our histopathological findings further confirmed that *E. arvense* treatment significantly restored pancreatic islet structure in MTZ-exposed rats. This supports previous reports revealing that *E. arvense* extract ameliorated pancreatic lesions in diabetic rabbits [47] and increased islet size and β-cell numbers in diabetic rats [48]. These outcomes reinforce the potential of *E. arvense* as a protective agent against MTZ-induced pancreatic injury.

Despite its antioxidant activity, olive leaf extract alone did not provide complete protection against pancreatic damage. Histopathological analyses revealed residual lesions in MTZ-induced pancreatitis even with olive leaf extract treatment, consistent with earlier findings suggesting incomplete protection by olive oil in experimental pancreatitis models [49].

## 5. Conclusions

This study demonstrates that *Equisetum arvense* (horsetail) ethanol extract provides a marked protection against metronidazole-induced pancreatic injury in rats, surpassing the effects of *Olea europaea* (olive) leaf extract. The protective efficacy of *E. arvense* was evidenced by normalization of pancreatic enzyme and glucose levels, enhancement of antioxidant defenses, downregulation of inflammatory mediators, and restoration of pancreatic architecture. These effects are largely mediated through activation of the PI3K/Akt/Nrf2 pathway and suppression of apoptotic signaling.

Despite its promising outcomes, the study was limited to a single sex and fixed dose levels, without evaluating the pharmacokinetic behavior of the active constituents. Future research should therefore investigate dose optimization, sex-related responses, and the specific bioactive compounds responsible for the observed protective effects.

Collectively, the results suggest that *Equisetum arvense* is a potent natural therapeutic candidate for alleviating drug-induced pancreatitis through antioxidant, anti-inflammatory, and anti-apoptotic mechanisms. These findings pave the way for advanced molecular and translational studies to validate its clinical applicability in both veterinary and biomedical contexts.

## Figures and Tables

**Table 1:** Primer sequence used for real-time PCR.

Gene Symbol	Gene Description	Accession Number	Primer Sequence
*Pik3ca*	phosphatidylinositol-4,5-bisphosphate 3-kinase, catalytic subunit alpha	NM_133399.3	F: 5′-TAGTGTCCGGGAAAATGGCT-3′ R: 5′-GGCATGCTCTTCGATCACAG-3′
*AKT*	AKT Serine/Threonine Kinase 1	NM_033230.3	F: 5′-CTGCCCTTCTACAACCAGGA-3′ R: 5′-GTGCTGCATGATCTCCTTGG-3′
*Nrf 2*	Nuclear factor, erythroid 2-like 2	NC_005102.4	F: 5′-GGCCCTCAATAGTGCTCAG-3′ R: -5′-TAGGCACCTGTGGCAGATTC-3′
*TNF-α*	tumor necrosis factor alpha	NM_012675.3	F: 5′-ACACACGAGACGCTGAAGTA-3′ R: 5′-GGAACAGTCTGGGAAGCTCT-3′
*IL-1β*	interleukin-1 beta	NM_031512.2	F: 5′- TTGAGTCTGCACAGTTCCCC -3′ R: 5′- GTCCTGGGGAAGGCATTAGG -3′
*GAPDH*	Glyceraldehyde3-phosphate dehydrogenase	NC_005103.4	F: -5′-ACCACAGTCCATGCCATCAC-3′ R: -5′-TCCACCACCCTGTTGCTGTA-3′

## Data Availability

All data generated and/or analyzed during this study are available from the corresponding author upon reasonable request. Due to institutional policy and ethical constraints, the raw data cannot be made publicly available but can be shared upon request with appropriate justification.
